# The Role of Oral and Gut Microbiota in Bone Health: Insights from Bacterial Extracellular Vesicles

**DOI:** 10.3390/microorganisms13102254

**Published:** 2025-09-25

**Authors:** Ping Liang, Xuanyu Chen, Zhikang Su, Yunlin Luo, Tao Wang, Jiang Li, Lvhua Guo, Tao Luo

**Affiliations:** 1 Guangdong Engineering Research Center of Oral Restoration and Reconstruction, Guangzhou Key Laboratory of Basic and Applied Research of Oral Regenerative Medicine, Department of Prosthodontics, Affiliated Stomatology Hospital of Guangzhou Medical University, Guangzhou 510182, China; 2021119035@stu.gzhmu.edu.cn (P.L.); luoyunlin@stu.gzhmu.edu.cn (Y.L.); maggiewinner4ever@163.com (T.W.); ljiang@gzhmu.edu.cn (J.L.); 2 Department of Basic Oral Medicine, School and Hospital of Stomatology, Guangzhou Medical University, Guangzhou 510180, China; 1026152150cxy@gmail.com (X.C.); 2024140388@stu.cqmu.edu.cn (Z.S.); 3 Department of Stomatology, Chaozhou Central Hospital, Chaozhou 521021, China; 4 Department of Basic Oral Medicine, Affiliated Stomatology Hospital of Guangzhou Medical University, Guangzhou 511436, China

**Keywords:** bacterial extracellular vesicles, microbiota, dysbiosis, osteoporosis, osteoarthritis, neuroinflammation

## Abstract

Bone health is critically influenced by the oral and gut microbiota, which are among the largest microbial reservoirs in the human body. These microbiota play essential roles in maintaining bone mass through immune modulation, metabolite production, and nutrient resorption. Recent observations have underscored that extracellular vesicles (EVs) derived from oral and gut microbiota may circulate to the brain and bone marrow, suggesting their integral roles in the gut–brain–bone axis and oral–brain–bone axis. This review outlines the current research status of bacterial extracellular vesicles (BEVs), including their biogenesis, classification, structural features, and cargo composition, with emphasis on factors influencing cargo heterogeneity and the consequences of cellular uptake and presentation. Oral-microbiota-derived BEVs and their cargo associated with bone health are highlighted, along with recent evidence linking BEVs to systemic dis-eases and the potential integration into the oral–gut–bone axis. Preclinical animal studies on BEV dosage, routes of administration, and disease models are summarized, together with the limitations of current approaches and strategies for engineering BEVs. Finally, an overview of translational applications and future therapeutic prospects is provided, aiming to advance the understanding of BEVs as innovative tools for the treatment and prevention of bone-related diseases.

## 1. Introduction

The oral–gut microbiome are two complex microbial ecosystems in the human body that can provide important functions for the host [1,2]. The oral cavity, a highly dynamic microbial environment, constantly faces complex and ever-changing challenges from external factors. It is the first organ to encounter exogenous microbes, allergens, and antigens before they reach the gastrointestinal tract (GIT) [3]. The gut microbiota (GM) contributes to the human physiological development and maintenance, including immune system establishment, nutritional digestion, and defense against the colonization of pathogen [4,5]. Both oral and gut microbial communities establish early in life. For instance, *Streptococcus salivarius* is among the first colonizers of both the oral cavity and gut after birth, contributing to the formation of a stable and individualized microbial profile that resembles a “microbial fingerprint,” supporting host–microbe symbiosis and protection against invading species [6]. However, perturbations caused by environmental insults or host genetic factors can disrupt this equilibrium, leading to dysbiosis, which is broadly defined as structural and functional imbalances in microbial communities [7]. In the oral cavity, dysbiosis is characterized not simply by reduced diversity but by shifts in microbial abundance and enrichment of pathogenic taxa, resulting in tissue destruction and persistent inflammation. In the gut, dysbiosis often manifests as decreased microbial diversity. Importantly, microbial functions are context-dependent and cannot be rigidly categorized as “beneficial” or “harmful.” For example, *Streptococcus mutans* is a well-recognized cariogenic pathogen due to its acidogenicity and biofilm-forming capacity; yet, EVs derived from *S. mutans* have recently been shown to promote skin wound healing via tRNA cargo, highlighting condition-specific beneficial roles [8].

Oral pathogens may also exert systemic effects through multiple routes, including hematogenous dissemination, translocation via the digestive tract, release of metabolites, or modulation of systemic immunity, endocrine networks, and the nervous system [9]. These processes can directly or indirectly influence gut microbial composition. Conversely, although less common, gut dysbiosis can also affect the oral microbiome. For example, both animal studies and clinical observations suggest that inflammatory bowel disease (IBD) is associated with increased abundance of pathogenic oral taxa and higher risk of periodontitis [10,11]. Such crosstalk is often mediated indirectly—for instance, through increased intestinal permeability and systemic immune activation—rather than direct colonization.

One important strategy by which bacteria influence their environment is the release of BEVs, nanosized bilayered particles universally produced by both Gram-negative and Gram-positive species. These vesicles encapsulate a diverse repertoire of biomolecules—including proteins, nucleic acids, lipids, metabolites, and toxins—that mirror their bacterial origin and confer substantial functional diversity [12]. Because vesicles can arise through multiple biogenetic routes, the resulting subtypes vary in composition and activity, generating heterogeneity even within a single bacterial species [13]. Functionally, BEVs are pivotal mediators of microbial ecology and host interaction. They enable the export and dissemination of bioactive molecules, protect bacteria against environmental threats such as antibiotics and phages, and contribute to interbacterial competition and biofilm development [14]. In parallel, they facilitate the transfer of virulence factors to host cells and modulate host responses by inducing inflammation or immune tolerance [14,15]. Recent studies suggest that BEVs provide a unique means for the oral–gut microbiota to influence systemic bone homeostasis. On one hand, commensal-derived BEVs may enhance bone formation by modulating immune responses or delivering beneficial metabolites; on the other, pathogen-derived BEVs may disrupt bone remodeling by promoting inflammation or carrying osteoclastogenic factors. Thus, BEVs function as a “double-edged sword”: a potential therapeutic avenue to improve bone health, but also a pathway for microbial dysbiosis to drive bone disease.

Consistent with this, BEVs have been detected in human blood, feces, and other biological fluids, underscoring their potential utility as disease biomarkers [16,17,18,19]. Because BEVs are capable of crossing biological barriers such as the intestinal and blood–brain barriers (BBB), they may act as systemic mediators that regulate the oral–gut axis and, through the gut–brain axis, synergistically contribute to pathogenic processes affecting long bones. This review systematically searched the PubMed, Web of Science, and Embase databases from their inception to 20 August 2025, using combinations of the following terms: “bacterial extracellular vesicles,” “oral gut microbiota,” “bone disease,” and “gut–brain axis.” Eligible publications included original research articles, reviews, and preclinical or clinical trial reports, while meeting abstracts and case reports were excluded. The present review specifically focuses on: (1) the contributions of oral and gut microbiota to bone health; (2) the mechanisms by which microbiota-derived BEVs mediate microbe–microbe and host–cell interactions, with particular emphasis on cargo selectivity and translocation across biological barriers; and (3) the roles of BEVs in osteoarthritis and other bone-related diseases, alongside their translational potential as therapeutic agents. Finally, unresolved challenges and future directions are discussed, with the goal of providing new insights into how oral- and gut-derived BEVs regulate bone remodeling and offering perspectives for the prevention and treatment of skeletal disorders.

## 2. Oral–Gut Bacteria Involved in Regulating Bone Homeostasis

**Figure 1:**
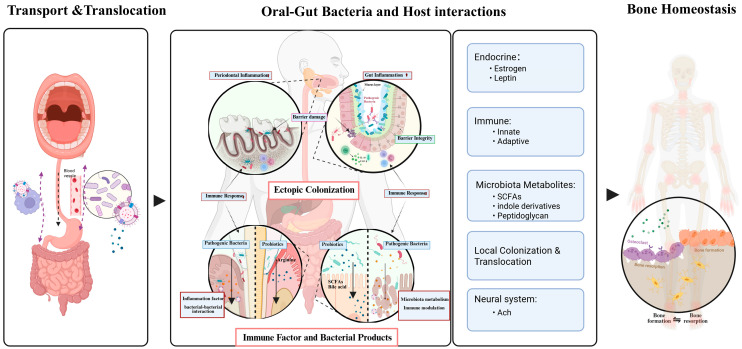
Schematic illustration of the oral–gut–bone axis mediated by BEVs. Oral and gut bacteria secrete BEVs that can translocate across epithelial and vascular barriers, disseminating systemically. These vesicles interact with host cells and shape immune responses through multiple mechanisms, including periodontal and gut inflammation, barrier integrity disruption, ectopic colonization, and the release of microbial metabolites (e.g., short-chain fatty acids (SCFAs), indole derivatives, peptidoglycan (PG)). Together, these processes modulate osteoclast and osteoblast activity, ultimately determining bone homeostasis through the balance of bone resorption and bone formation. Created in Biorender (2025).

As our exploration of microorganisms deepens, research on the role of oral–gut bacteria extends beyond their growth sites. Bacterial involvement has been observed in various systemic diseases, including Alzheimer’s disease (AD) [20], rheumatoid arthritis (RA) [21], cardiovascular disorders [22], systemic bone loss [23], IBD [24], and liver cirrhosis [25]. Maintaining dynamic balance in bacterial populations is a hallmark of host health. When bacterial abundance, diversity, or uniformity changes, local tissue homeostasis and normal immune responses can be disrupted [26,27]. Additionally, bacteria participate in metabolic processes in distant organs through various mechanisms. Recent reviews suggest that alterations in bacterial homeostasis can impact the dynamic balance of bone mass, affecting processes such as growth, repair, aging, and resorption [28,29,30]. Current evidence highlights three major pathways—direct colonization, modulation of local immune responses, and the action of bacterial components and metabolites—as illustrated in Figure 1.

### 2.1. Extracorporeal Implantation

In adults, the daily swallowing of saliva exceeds 1000 mL. Bacteria in the oral cavity can migrate to the intestines through swallowing, leading to an imbalance in GM and affecting the organs of the digestive system [31,32]. In patients with IBD, inflammatory gut epithelium and feces contain more microbial nucleic acid from oral sources [33], such as fusobacteria, which are almost undetectable in the gut epithelium of healthy individuals [34]. Bacteria can enter the systemic circulation through the oral mucosal epithelium and gut barrier, particularly when the integrity of the mucosal barrier is compromised. For example. periodontal tissue destruction resulting from periodontal disease serves as a reservoir for bacteria [35], while increased gut permeability has been observed in systemic diseases such as diabetes and gut inflammation. This heightened permeability facilitates the translocation of bacterial and microbial products into the systemic circulation [25]. Bacterial colonization in remote areas has been indicated such organs: (1) bacteria enter the liver via the portal vein, they can act on liver cells and stellate cells, leading to the further production of inflammatory mediators and the development of liver cirrhosis [25]. (2) Bacteria entering the circulation trigger an excessive immune response, leading to inflammation and the formation of atherosclerotic plaques within the cardiovascular arteries. This is substantiated by the extraction of live bacteria from these plaques [36,37]. (3) Bacteria colonized the lung tissue of patients with mild to moderate chronic obstructive pulmonary disease (COPD), exacerbating the progression of COPD [38]. (4) The entry of bacteria into the bloodstream and subsequent systemic inflammation may also lead to the development of bone diseases [39]. Apart from periodontal pathogens causing bone resorption through pathways [40,41,42,43], such as periodontal pockets, dentin–pulp complexes, and periapical colonization, research on non-invasive bacterial colonization within the whole body’s bones remains limited. Steinwender et al. discovered that in neonatal mice with impaired gut function, there is a risk of bacterial translocation and bone infection [44]. Chukkapalli et al. found that periodontal pathogens colonize the temporomandibular joint in mice, exacerbating disease progression, possibly related to hematogenous infections [45]. Other studies on bacteria colonizing bones are often associated with orthopedic surgery [46] and invasive dental procedures [47]. Bacteria participate in systemic bone homeostasis through immune regulation, shedding products, and metabolic byproducts, which may have significant implications.

### 2.2. Local Immune Factor Propagation

The relationship between oral–gut microbiota and bone immunity is closely intertwined. The gut microbiome regulates the proliferation and activation of helper T cells, modulating bone growth by dynamically balancing interactions between T cells and growth factors such as transforming growth factor-beta (TGF-β) and insulin-like growth factor 1 (IGF-1) [48]. Similarly, oral microbiota also promote bone remodeling during skeletal development through similar pathways, preventing adverse bone structural changes caused by excessive mineralization [49].

The pathogenicity of oral–gut bacteria is mainly attributed to microbial dysbiosis and barrier dysfunction. Barrier failure enhances exposure of submucosal immune cells to microbial products, leading to their activation and heightened release of pro-inflammatory mediators. This local immune overactivation subsequently drives systemic immune alterations [50]. For instance, in diabetic and obese mice models, impaired gut barrier function leads to increased expression of local inflammatory factors, such as IL-1β, IL-6, and tumor necrosis factor-alpha (TNF-α), which then spill over into the systemic circulation [51]. Similarly, in patients with periodontal disease, increased expression of pro-inflammatory mediators (IL-1, IL-6, and C-reactive protein) at the site of the lesion drives alveolar bone resorption and triggers systemic inflammation when these mediators enter the bloodstream. Furthermore, the sensitivity of bone marrow precursor cells to inflammatory signals skews their differentiation toward osteoclasts within the bone marrow lineage. These observations underscore the interconnectedness of local bone loss and systemic bone metabolism [50,52]. Therefore, the process of local immune modulation by oral–gut bacteria significantly contributes to overall immune regulation of bone metabolism [29,53].

### 2.3. Bacterial Components and Metabolites

The structural components and metabolites of bacteria are key activators of bone immune responses and metabolism [54]. The GM helps maintain homeostasis and balance [53] between host immune tolerance to bacteria and immunity to pathogens through metabolites obtained from modifying host products and processing digested food, such as SCFAs, bile acids, ammonia, etc. [55]. SCFAs lower the pH value in the gut, improve the metabolism of metal ions, increase the absorption of calcium, magnesium, and iron to regulate bone development, and inhibit the generation of osteoclasts by secreting IL-4 and IL-10. It has also been reported that probiotics or probiotic derivatives, such as ammonia produced by the decomposition of arginine, maintain the pH value in the oral cavity, which helps prevent oral diseases such as caries and mucosal diseases [55,56,57].

BEVs are intricate complexes comprising bacterial structural components and metabolites, serving as vehicles for the long-distance delivery of bacterial cargo molecules. They are messengers that regulate microbial symbiosis and competition, protect bacteria from antibodies and antimicrobial peptides (AMPs) [58], and are key participants in bacterial-host communication. BEVs secreted by gut bacteria (including probiotics) effectively prevent and treat gut inflammation [59], protect the gut barrier, regulate microbial homeostasis, and can also be transferred to distant bone tissues to regulate bone metabolism by regulating the host immune system and gut barrier [60,61]. Conversely, it is worth noting that BEVs from oral–gut pathogens transferred to bone tissues induce or exacerbate pathological changes in bone diseases [53,59]. Components of BEVs may modulate the gut microbiota and its metabolites, which in turn influence how cargo is selected and modified during BEV biogenesis. This process will be discussed in the following section. Furthermore, gut microbiota can sense nutrient and modulate both local and systemic hormone levels [62]. These findings highlight a potential mechanism through which BEVs may systemically regulate bone metabolism via interactions with the gut microbiota.

## 3. Characteristics and Communicative Functions of Oral–Gut BEVs

The secretion of BEVs requires de novo membrane synthesis, representing a seemingly energy-intensive strategy. However, this highlights the evolutionary advantage of microbes in deploying BEVs as adaptive tools to interact with and remodel their environment. Understanding the underlying logic of BEV release and the mechanisms by which they interact with host cells is essential for clarifying their roles in disease pathogenesis and in maintaining microbiota stability. Such knowledge may also facilitate the exploitation of BEVs for therapeutic applications or preventive strategies.

### 3.1. Occurrence, Composition, and Transport of BEVs

#### 3.1.1. Production of BEVs

Different bacterial species have distinct pathways to generate EVs, with mechanisms in Gram-negative bacteria being comparatively well characterized. Three major types of vesicles have been described: the classic outer membrane vesicles (OMVs) produced by the bubbling of living cells, outer–inner membrane vesicles (OIMVs) and explosive outer membrane vesicles (EOMVs) produced by cell rupture triggered by endolysins, and OIMVs formed by the transient degradation of PG by autolysins [12,63,64]. The biogenesis mechanism of BEVs in Gram-positive bacteria is not as clear as that in Gram-negative bacteria, and the process of production and release is still controversial [61,63]. Currently, there are three main hypotheses for the mechanism of BEVs secretion through the cell wall of Gram-positive bacteria [65]: (1) vesicle accumulation between the cytoplasmic membrane and the thick PG wall generates pressure until release; (2) enzymatic degradation of PG creates pores that allow vesicle passage; and (3) protein channels or structural cables guide vesicles across the cell wall. Recent studies in archaea have shown that EVs from *Methanobrevibacter smithii* accumulate in the periplasmic space and are released into the extracellular milieu through localized disruption of the rigid cell wall, a process reminiscent of vesicle passage across Gram-positive walls [66]. Advanced imaging techniques, including cryo-electron tomography (cryo-ET) and super-resolution stochastic optical reconstruction microscopy (STORM), are anticipated to provide deeper mechanistic insights into EV biogenesis in Gram-positive bacteria, where thick PG layers present unique structural challenges.

Vesicle release is not merely a passive byproduct of physical pressure but is also under genetic control as evidenced by studies. In Gram-negative bacteria, for example, outer membrane–PG anchoring proteins such as OmpA and Lpp in *Escherichia coli* (*E. coli*) play key stabilizing roles; deletion of these genes significantly increases OMV release [67]. Similarly, in archaea, vesicle production in *Haloferax volcanii* has been shown to depend on a small Ras-like GTPase (ArvA) and its operon partners, providing direct evidence that prokaryotes can employ conserved membrane-remodeling machineries reminiscent of eukaryotic vesicle trafficking [68]. This suggests that bacteria can actively regulate vesicle output by modulating anchoring proteins. In mammalian cells, EV formation is also governed by genetic factors, with the endosomal sorting complex required for transport (ESCRT) machinery representing a canonical pathway. Together, these examples illustrate that EV biogenesis is subject to tightly controlled genetic regulation.

**Figure 2:**
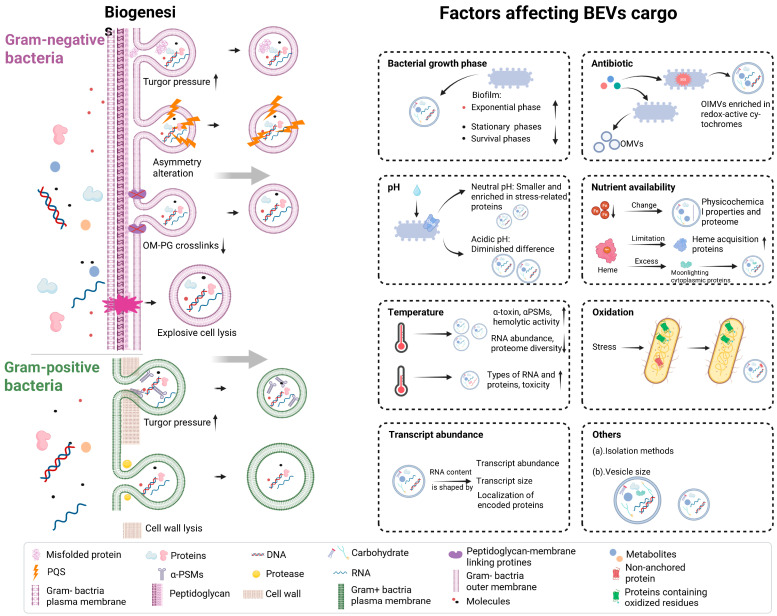
Biogenesis of BEVs and key factors shaping their cargo. Gram-negative and Gram-positive bacteria utilize distinct pathways for vesicle release, while growth conditions, environmental stressors, and isolation methods determine the molecular heterogeneity of BEVs. Created in Biorender. (2025).

However, studies on the regulatory mechanisms of Gram-positive bacterial proteins remain limited. Autolysins, which are self-encoded PG hydrolases, have been implicated in vesicle release. For example, *S. mutans lytF* mutants exhibit altered EV production [69], and in *B. subtilis*, multiple autolysin genes—including *LytC*, *LytD*, *LytE*, and *LytF*—have been shown to influence EV secretion [70]. Importantly, distinct biogenetic routes directly influence cargo selectivity—for example, OMVs formed by blebbing exclude cytoplasmic material, whereas OIMVs and EOMVs package both cytoplasmic and membrane-derived cargo (see also Section 3.1.2 and Figure 2). These findings suggest that Gram-positive species may also rely on genetic regulatory networks, in addition to the physical constraints of thick PG walls and pressure-driven release, to control EV biogenesis.

#### 3.1.2. Composition of BEVs

The composition of BEVs is different from that of their parent bacterial cells, with varying degrees of enrichment observed for individual components within BEVs, but their composition differs between Gram-negative and Gram-positive. Vesicles from Gram-negative species typically contain PG fragments, membrane proteins (MPs), lipoproteins, lipopolysaccharides (LPS) or lipooligosaccharides (LOS), and phospholipids, whereas Gram-positive vesicles harbor PGN, MPs, lipoproteins, and lipoteichoic acid (LTA) but lack LPS/LOS.

The membrane protein components enriched in BEVs are influenced by the differential affinity of membranes to various proteins, with outer membrane (OM) integration proteins being preferentially retained. BEVs can also encapsulate diverse compounds, including cytoplasmic proteins, secreted proteins, and nucleic acids (DNA and RNA) [71]. The specific enrichment processes that govern cytoplasmic loading during these distinct vesiculation pathways remain poorly understood and require further investigation [14,72]. Interestingly, many bacteria appear to preferentially release certain components via BEVs rather than retain them within the cell, a phenomenon that may reflect an evolutionarily conserved survival strategy. In *Porphyromonas gingivalis*, virulence factors such as gingipains and adhesins are selectively enriched in OMVs—often at concentrations significantly higher than in the parent bacterial cell—highlighting EVs as potent mediators of pathogenesis via concentrated toxin delivery [73]. In addition, at different growth stages of bacteria, the physicochemical properties and functionality of BEVs will be different. For example, late-log phase BEVs and stationary phase BEVs of *P. gingivalis* have more significant pathogenicity in periodontal disease, even almost equal to the pathogenic effect of the parent bacteria [74]. Variations in cargo abundance or structure endow BEVs with unique regulatory roles. For instance, BEV derived from *P. gingivalis* enrich virulence *P. gingivalis* proteinase, facilitate their protection against degradation during long-distance delivery, thereby prolonging their activity [75,76]. This kind of BEVs carrying virulence is not just a simple pathogenic effect, it may be related to the host’s early establishment of immune tolerance to the microbiota [45].

BEVs are not only diverse in composition, but also selectively package cargo based on multiple factors, reflecting bacteria’s ability to adapt to and interact with their environment. This capacity to modulate cargo allows bacteria to enhance survival and adaptability by coordinating or competing across diverse environments. Oral pathogens such as *P. gingivalis* (gingipains), *A. actinomycetemcomitans* (leukotoxin), and *Fusobacterium nucleatum* (*F. nucleatum*) (FadA) preferentially enrich virulence factors in their vesicles, thereby aggravating host tissue destruction and immune dysregulation. In contrast, commensal species such as Bacteroides employ OMVs to selectively package glycan-degrading enzymes that facilitate nutrient acquisition. For example, proteomic analyses of *Bacteroides thetaiotaomicron* OMVs demonstrated that glycosidases and proteases are enriched through a negatively charged lipoprotein export signal (LES) motif, enabling these vesicles to act as extracellular “digestive units” that expand polysaccharide utilization. Moreover, the composition of bacterial EVs can vary substantially depending on the strain and culture conditions, reflecting the ability of bacteria to actively tailor EV cargo in response to environmental cues and fine-tune their interactions with the host [77]. The following factors are recognized as major determinants of selective cargo packaging, as depicted in Figure 2: (1) Bacterial growth phase. EV secretion varies across growth stages. In *Pseudomonas aeruginosa*, EVs derived from biofilms promote biofilm expansion during the exponential phase, while vesicles secreted during stationary or survival phases may reduce biofilm growth, reinforcing their role in cell-to-cell communication [78]. (2) Antibiotics. Sub-minimum inhibitory concentration (sub-MIC) antibiotics can markedly remodel EV cargo. For example, in *Geobacter sulfurreducens*, ciprofloxacin triggers SOS response and prophage induction, leading to the release of OIMVs enriched in redox-active cytochromes, which enhance Fe(III) reduction capacity, whereas ampicillin primarily induces classical OMVs through outer-membrane blebbing [79]. Thus, sub-MIC antibiotics act as external regulators of vesicle composition and microbial energy metabolism. (3) pH and specific regulators. In Gram-positive *Streptococcus mutans*, the OpuB transporter modulates vesicle size, lipid composition, and proteomic content. Under neutral pH, the *opuB* mutant secreted significantly more and smaller vesicles enriched in stress-related proteins such as DNA repair factors, whereas under acidic pH these differences diminished. Together, pH and OpuB regulate MV biogenesis and cargo packaging, enhancing adaptation to cariogenic acidic niches [80]. Furthermore, the growth of *Helicobacter pylori* under acidic conditions leads to the production of fewer and smaller OMVs, with altered protein enrichment profiles. In contrast, under near-neutral pH, OMVs are enriched in VacA and the putative β-lactamase HcpE, suggesting their potential involvement in pathogenic processes [81]. (4) Nutrient availability. Iron and heme strongly influence OMV cargo. In *Neisseria gonorrhoeae*, iron limitation alters OMV proteome and physicochemical properties [82]. In *P. gingivalis*, heme limitation upregulates heme acquisition proteins, whereas heme excess induces a heme efflux system and preferential packaging of moonlighting cytoplasmic proteins into OMVs [83]. These findings underscore nutrient-driven plasticity in EV cargo. (5) Temperature. In *Staphylococcus aureus* (*S. aureus*), subtle temperature changes markedly alter EV content. At 40 °C, vesicles are enriched in virulence factors such as α-toxin and αPSMs, enhancing hemolytic activity but reducing RNA abundance and proteome diversity. At 34 °C, vesicles carry more diverse proteins and RNAs and exhibit higher cytotoxicity toward macrophages [84]. The temperature effect may be mediated by changes in membrane fluidity: high temperatures increase fluidity and promote vesicle budding, whereas low temperatures decrease fluidity and reduce vesiculation [84]. (6) Oxidation. In *E. coli*, OMV cargo selectivity is largely determined by whether proteins are stably tethered to the cell envelope and whether they contain oxidizable residues, with integral MPs being retained in the OM and untethered or oxidized proteins preferentially exported into OMVs under stress [85]. (7) Transcript abundance and RNA selectivity. EV RNA content is shaped by transcript abundance, size, and the localization of encoded proteins. In *Lactobacillus johnsonii N6.2*, vesicle-associated RNAs were enriched in highly expressed, smaller transcripts encoding membrane-bound or secreted proteins. These RNAs elicited RNA-sensing immune pathways in host cells, emphasizing functional consequences of RNA selectivity [86]. The diverse molecular cargo of BEVs not only shape host–microbe interactions but also mediate complex communication between bacterial species, which will be discussed in the next section.

In addition, EV isolation methods can influence cargo composition, and vesicle size itself is another determinant, typically ranging from 20 to 400 nm [87]. Larger vesicles exhibit greater cargo capacity and distinct loading efficiencies compared with smaller counterparts. Although consensus guidelines such as Minimal Information for Studies of Extracellular Vesicles provide recommendations on isolation strategies and purity standards, this heterogeneity underscores the need to interpret functional studies with caution [88].

The ability of BEVs to selectively package cargo represents a promising strategy, as it allows the reduction in toxic components while enabling the enrichment of immunomodulatory molecules that promote tolerance and host protection. By controlling culture conditions or applying bioengineering strategies, BEVs can be tailored for clinical applications: (i) removing or reducing toxic cargo (e.g., LPS, toxins) to improve safety; (ii) enriching beneficial molecules (e.g., immunoregulatory proteins, metabolites, or therapeutic agents) to enhance efficacy; and (iii) ensuring reproducibility and consistency across production batches. Synthetic biology approaches have been used to engineer *E. coli*-derived OMVs to carry heterologous antigens or drugs, taking advantage of vesicles’ natural capacity to package specific proteins or nucleic acids [89]. Thus, selective cargo packaging, once standardized, may transform BEVs into powerful tools for vaccines, drug delivery, and the treatment of inflammatory and metabolic diseases.

#### 3.1.3. Transport of BEVs

Recent large-scale human microbiome studies revealed that oral microbes can transmit to and colonize the large intestine, establishing the oral cavity as an endogenous reservoir for gut microbial strains [90]. However, this remains observational evidence, and longitudinal follow-up with oral disease stratification is needed. Direct evidence of oral bacteria entering the gut largely comes from animal studies. For example, *F. nucleatum* introduced to induce periapical periodontitis in mice was detected in the colon within two weeks, accompanied by alterations in GM composition [91]. Given the highly acidic environment of the stomach, direct survival and EV release of oral bacteria along the GIT may be limited. Nevertheless, it cannot be excluded that oral bacteria enter the gut in biofilm aggregates to resist low pH, or under conditions of impaired gastric acid or bile barrier function, where they can colonize and release EVs [92]. Indeed, *P. gingivalis* and its OMVs have been detected in the GM of patients with IBD. The hydrophobic properties of BEVs protect encapsulated cargo from degradation during long-distance transport and facilitate their uptake by host epithelial and endothelial cells, thereby contributing to inflammation in distal organs such as neuroinflammation [93,94] and bone loss [21,95]. Moreover, the nanoscale size of BEVs allows them to penetrate leaky vasculature, enter the circulation, or cross epithelial and endothelial barriers such as the intestinal epithelium and the BBB.

Crossing the intestinal barrier involves multiple layers: (1) Chemical barrier: BEVs can neutralize AMPs [96] or impair commensal growth [97]; (2) Physical barrier: tight junctions formed by proteins such as occludin and E-cadherin. Pathogenic BEVs (e.g., *P. gingivalis* OMVs) can downregulate tight-junction proteins and compromise epithelial integrity [98]; immune barrier: epithelial cells recognize BEVs via pattern-recognition receptors such as TLRs (e.g., TLR4) and intracellular receptors such as NOD1, leading to cytokine release [99]. After transcytosis across intestinal epithelial cells, BEVs reach the lamina propria, where they are internalized by resident immune cells, including macrophages and dendritic cells (DCs). Notably, EV uptake by epithelial or immune cells is not merely part of barrier clearance but may also facilitate trans-barrier transport. During cell death processes (apoptosis, necrosis, pyroptosis) or transcytosis, EV cargo can be further released to the opposite side of the barrier [100]. This dual role underscores potential therapeutic applications, such as exploiting commensal-derived EVs to reinforce barrier function by promoting occludin expression or reducing pathogen adhesion.

With respect to the BBB, systematic reviews have suggested that bacterial EVs can indeed cross, though the precise mechanisms remain poorly defined. Most experimental data derive from in vitro permeability models or zebrafish in vivo studies [101,102]. In mammalian EV research, known transport mechanisms include transcytosis [103], receptor-mediated transport [102], and transient disruption of tight junctions. However, current models are largely based on single-layer systems (e.g., transwell, organ-on-chip) and do not fully recapitulate the neurovascular unit. Direct in vivo evidence for gut-derived BEVs crossing the BBB is still lacking [104].

### 3.2. BEVs as a Communication Medium

BEVs serve as both protective and communicative vehicles, allowing bacteria to persist under hostile host conditions. For instance, EVs secreted by gut commensals that contain low amounts of LPS can elicit a state of trained immunity, thereby exerting probiotic and immunoregulatory effects [63]. In contrast, the membrane structure of Pg confers resistance to host RNases, enabling its vesicles to travel long distances, undergo targeted uptake by host cells, and consequently contribute to neurological dysfunction, exacerbate periodontitis, or even adaptively target erythrocytes to optimize nutrient acquisition [105]. When *Pseudomonas aeruginosa* is exposed to H_2_O_2_, OMV production increases, with catalase KatA selectively packaged into OMVs to mitigate oxidative stress [106]. Similarly, *Helicobacter pylori* vesicles attenuate extracellular oxidative stress during host respiratory bursts [64]. Such evolutionarily conserved strategies demonstrate how bacteria not only adapt to their surroundings but also actively reshape them.

#### 3.2.1. Communication Between Bacteria

The relationship between bacteria is complex, dynamic, and bidirectional. Bacteria establish and maintain cooperative, symbiotic, and competitive relationships among different genera through quorum sensing (QS), biofilm formation, nutrient acquisition, defense, and other means [95,104,107,108,109]. By packaging signaling molecules, enzymes, and even genetic material, BEVs enable cooperation and competition within microbial communities.

QS serves as a chemical signaling method that orchestrates the behaviors of bacterial communities. It plays a pivotal role in the secretion processes of BEVs from parental bacteria. Proteins related to QS are also released through these BEVs, which are instrumental in the nutrient acquisition processes of bacteria. BEVs are carriers of essential nutrients, including iron ions, vitamins, amino acids, and carbon metabolic components. Furthermore, they transport critical hydrolytic enzymes enabling certain bacteria to access polysaccharides [14,107]. The role of BEVs in environmental stress mitigation involves their content, such as catalase, which degrades reactive oxygen species secreted by host cells. They also contain nucleic acids, including genes for antibiotic resistance; however, the mechanisms of nucleic acid uptake and function in recipient cells remain poorly understood. Additionally, BEVs can function as protective “bait” in hostile environments by binding to bacteriophages and AMPs, thus enhancing the survival of bacterial communities. For instance, BEVs secreted by *P. gingivalis* can bind to chlorhexidine and extend their protective effect to the biofilm they are in [95].

BEVs also constitute a significant component of the bacterial biofilm matrix, contributing to the biofilm’s structural integrity and functional enhancement [109]. For example, *P. gingivalis* BEVs improve the aggregation and adhesion of diverse oral microbes within dental plaque biofilms [110]. Similarly, BEVs from *Pseudomonas* carry glycoside hydrolases that can break down various polysaccharides [95]. EVs secreted by *Streptococcus mutans* may serve as a mechanism of bacterial competition and represent an important dysbiotic factor during the early stages of cariogenic biofilm establishment [111]. In contrast, EV production by *P. gingivalis* appears to be highly dependent on strain-specific genotypes, and clinically, dental biofilms are likely to harbor multiple EV subtypes derived from diverse *P. gingivalis* lineages.

Moreover, BEVs contain bioactive molecules that exhibit antifungal and antibacterial properties, which are crucial in bacterial competition and survival. For instance, *Bacillus* and *Staphylococcus* release bioactive compounds and lytic enzymes through their BEVs to eliminate competing genera. BEVs from *P. gingivalis* can suppress the adhesion-related proteins FadA and FomA of *F. nucleatum* diminishing its ability to adhere to and invade epithelial cells. Additionally, BEVs from *Clostridium butyricum* significantly reduce the abundance of bacterial pathogens such as *E. coli* and *Shigella flexneri* [112].

#### 3.2.2. Interaction Between BEVs and Host Cells

BEVs engage with human host cells through various mechanisms, including receptor binding, membrane fusion, endocytosis, and phagocytosis. These interactions can profoundly affect cellular functions such as inflammation, apoptosis, necrosis, and autophagy, and also influence the efficiency of cellular uptake of BEVs [113]. The cargo within BEVs can activate a broad spectrum of innate immune responses in the host. This activation promotes cellular immune reactions and cell death via membrane and cytoplasmic pattern recognition receptors (PRRs), such as LPS/LOS or DNA/RNA that activate the TLR pathways, leading to an inflammatory response. Furthermore, the conserved structure of PG can initiate the cytoplasmic NOD pathways, enhancing antibacterial immunity and autophagy [111]. Additional components of BEVs can independently regulate host immunity and promote pathogenesis. For example, internalized endotoxins may induce cellular necrosis. Small interfering RNAs (siRNAs), outer membrane proteins (OMPs), and LPS can modulate host gene expression and immunity, facilitating pathological progression [114,115].

BEV uptake is highly cell-type and receptor dependent, co-determined by vesicle-intrinsic features (size, surface ligands, cargo) and the endocytic repertoire of the recipient. Professional phagocytes (macrophages, DCs) internalize BEVs via phagocytosis and micropinocytosis via various receptors [116]. For example. *P. gingivalis* OMVs enriched in gingipains engage pattern-recognition receptors TLR2 to enhance uptake and pro-inflammatory signaling in macrophages [117]; *Francisella tularensis* EVs are taken up by bone-marrow-derived macrophages predominantly via macropinocytosis, as supported by inhibitor studies [118]. These uptake events frequently culminate in downstream cellular outcomes, including inflammation, apoptosis, necrosis, autophagy, or secondary release of vesicular cargo into the extracellular milieu.

Non-phagocytic epithelial cells (e.g., intestinal or oral mucosa) use distinct pathways such as clathrin-mediated endocytosis (CME), caveolin/lipid-raft-dependent endocytosis, and macropinocytosis. For example, Jones observed that intestinal epithelial cells principally acquire Bt OMVs via dynamin-dependent endocytosis followed by intracellular trafficking to LAMP-1 expressing endo-lysosomal vesicles and co-localization with the perinuclear membrane [119].

BEV size further dictates uptake fate: smaller vesicles (<100 nm) typically enter epithelial cells via CME or lipid-rafts and may escape lysosomal degradation to release bioactive cargo into the cytosol; larger vesicles (>200 nm) are more prone to phagocytosis and lysosomal routing [120]. Surface ligands also confer targeting specificity: Bacteroides fragilis OMVs, enriched with polysaccharide A, preferentially target DCs to activate TLR2 and induce regulatory T cell responses [121]; *Bacteroides thetaiotaomicron* employs a negatively charged LES to sort glycosidases into OMVs, optimizing interaction with gut epithelium and possibly influencing their cellular uptake [84].

Thus, BEV uptake is choreographed by vesicle size, cargo, and ligand receptor compatibility across distinct cell types. From a therapeutic perspective, these mechanisms provide opportunities to optimize engineered BEVs for bone-targeted interventions. To prevent premature degradation, strategies can be designed to bias uptake toward caveolin- or lipid-raft-mediated pathways, which favor cytosolic delivery over lysosomal degradation, thereby enhancing the functional release of therapeutic cargo such as osteogenic miRNAs. Conversely, avoiding excessive uptake by phagocytes (e.g., macrophages) is essential to reduce clearance and preserve bioactivity; this can be achieved by surface modifications that inhibit phagocytosis or by local delivery systems. To improve osteoblast targeting, BEVs can be functionalized with bone-homing ligands (e.g., CXCR4 to exploit SDF-1/CXCL12 gradients, RGD peptides to engage integrins, or bisphosphonate groups to bind mineralized matrix), thereby enriching them in the bone microenvironment and promoting efficient uptake by osteogenic cells. In addition, engineered OMVs can be designed to exploit the Enhanced Permeability and Retention (EPR) effect, thereby facilitating their preferential accumulation at target sites [122].

## 4. Oral–Gut-Derived BEVs and Systemic Bone Diseases

The diversity and activity of gut bacteria significantly affect bone density and overall bone health, impacting both healthy individuals and those with disease. This substantial evidence primarily originates from studies involving microbiota transplantation in germ-free environments or/and antibiotic interventions. Gut microbes primarily regulate bone homeostasis by modulating the immune system, interacting with hormones, enhancing nutrient absorption, and producing key metabolites like fatty acids [49,123]. Additionally, recent research highlights a potential role for the gut–brain axis in bone regulation [124]. Microbial dysbiosis in the oral cavity leads to changes in the composition of dental biofilm, shifting the balance towards predominantly pathogenic bacteria. These bacteria and their byproducts then enter the circulatory system through both open and closed foci, potentially causing systemic bone diseases. Microbial metabolites and BEVs can penetrate the BBB, emphasizing their roles in neural signaling and immune modulation [125]. Recent evidence points to the role of BEVs as communicative agents between oral/gut bacteria and the host in regulating distal bone mass are described in the following paragraph.

### 4.1. The Potential Role of Gut BEVs and Osteoporosis

The relationship between gut microbiota and bone mass has been established over the past two decades. The initial observation was made by Di Stefano et al. in 2001, who found that small intestine bacterial overgrowth was associated with reduced bone mineral density [126]. Subsequent research in 2012 demonstrated that experimental colonization of bacteria in mice maintained in germ-free (GF) conditions could restore bone mass [127]. It is now understood that commensal bacteria contribute to gut defense and metabolic functions. The abundance of microbiota changes during aging or due to factors such as drug use, estrogen fluctuations, and dialysis, which usually contribute indirectly to bone homeostasis through metabolites. These interact with epithelial cells of the GIT and immune cells beneath the mucosal lymphoid tissue. However, when the barrier function is compromised due to estrogen deficiency or microbiota dysbiosis, on rare occasions, these bacteria may translocate and directly interact with cells [128].

Unlike bacteria, nanosized BEVs can easily traverse barriers such as those of the GIT and even the BBB, enabling direct interactions with cells. A recent study demonstrated that GM from children (CGM), as opposed to that from the elderly (EGM), prevents reductions in bone mass and strength in mice with ovariectomy (OVX)-induced OP [129]. The secretion of BEVs plays a crucial role in these bone-protective effects, as these vesicles can enter and accumulate in bone tissues, thereby mitigating adverse osteoporotic changes by enhancing osteogenic activity and reducing osteoclast formation. *Akkermansia muciniphila* (*A. muciniphila*), a common gut bacterium, directly corrects imbalanced bone metabolism and protects against OP, highlights the function of BEVs in mediating the beneficial effects of GM. Intriguingly, the benefits of *A. muciniphila* were negated when the secretion of its extracellular vesicles was inhibited, impairing the release of these vesicles [129]. Probiotic-derived BEVs have also been shown to modulate bone mass. BEVs from *Lactobacillus animalis* (*L. animalis*) mitigated the reduction in survival and growth times of human microvascular endothelial cells (HMEC) and bone lineage cells induced by glucocorticoids, and facilitated the formation of HMEC lumens and mineralized nodules in bone marrow stromal cells (BMSCs) [130]. However, it is important to note that the bone-promoting effects of probiotics are not necessarily aligned with those of their BEVs. For example, *L. rhamnosus* can inhibit bone loss by blocking the Wnt signaling pathway in bone lineage and endothelial cells [131], yet *L. rhamnosus* BEVs did not exhibit similar effects to the parent bacteria, and their anti-apoptotic effect on bone lineage cells and endothelial cells was not as pronounced as that of *L. animalis* EVs [130].

Decreased diversity in the gut microbiota is associated with heightened susceptibility to pathogenic bacteria, which can contribute to bone resorption through their BEVs. Wang et al. found that *Proteus mirabilis* (*P. mirabilis*) EVs induce mitochondrial apoptosis by regulating *miR 96-5 p/Abca 1* to inhibit the differentiation and function of osteoclasts. Although it moderately impairs the maturation of osteoblasts, it significantly improves bone loss in OP and RA animal experimental models [59]. Diaz-Garrido et al. observed that the immune response elicited by different strains of *E. coli* varies; BEVs from probiotic *E. coli* Nissle 1917 induced a balanced Treg/Th17 response, whereas commensal *E. coli* EVs predominantly prompted Tregs to secrete TGF-β and stimulated Th17 cells to produce IL-17 [45,132]. OMVs from *P. multocida* has been shown to induce macrophage TGF-β1 expression in vitro [133]. However, while IL-17 is considered to be a cytokine that induce bone resorption, the role of TGF-β is complex. Some studies have reported that TGF-β plays a positive role in RANKL-induced osteoclastogenesis [132,134,135]; other researchers believe that TGF-β has inhibitory or dual (inhibitory and stimulating) effects on RANKL-induced osteoclastogenesis [136,137,138], as well as the effect of TGF-β on the expression of other cytokines, such as the response of macrophages to TNF, regulating osteoclastogenesis [139]. Additionally, the function of Tregs is also regulated by microbial products such as SCFAs [140,141].

SCFAs are microbial metabolites that could influence the proliferation and differentiation of osteoblasts and osteoclasts by activating G protein-coupled receptors and inhibiting histone deacetylases. Although no studies have conclusively shown that BEV are the primary means for these bacteria to transmit SCFAs, they can modulate the bacterial composition and thereby indirectly affect SCFAs production. SCFAs modulate osteoclast differentiation and inducing Tregs to secrete factors that inhibit osteoclast formation or directly suppress osteoclast differentiation through inhibition of cell-to-cell contact [142]. In the bone marrow, SCFAs has been shown to promote the differentiation of Tregs, activates the Wnt signaling pathway to promote osteoblast formation, and reduces cell apoptosis [141]. *Bifidobacterium*, *lactobacillus* and other gut probiotics are the main producers of SCFAs in the gut. A decreased proportion of their microbiota may lead to a significant reduction in levels of butyrate, propionate, and other SCFAs [142]. Moreover, some research indicates that the effects of butyrate-producing *Clostridium* BEVs on gut inflammation are comparable to those of the parent bacteria [143,144]. In addition, SCFAs are one of the important components of BEVs. We can speculate based on this that during the aging process of the body [145,146], the protective effect of BEVs secreted by *bifidobacteria*, *lactobacilli*, and other bacteria involved in SCFAs metabolism on bones gradually decreases, which is also one of the potential causes of OP/bone loss. Additionally, the fatty acids in BEVs resemble the lipids isolated from bacterial cells such as *E. coli* [147]. These phospholipids have been shown to modulate the immune response through the TLR2–TLR1 signaling pathway, underscoring the complex interactions between BEVs, microbial metabolites, and host immunity [148].

### 4.2. The Potential Role of Oral BEVs and Osteoporosis

Animal models of GF and specific pathogen-free (SPF) mouse model indicate that commensal oral microbiota induces bone loss through the activation of T cells and DCs [149]. Dysbiosis in the oral microbiome often leads to the dominance of a single pathogen, which is linked to localized diseases of periodontal disease. The progression of periodontitis involves interactions between the subgingival biofilm, host cells, and environmental nutrients. However, research on the impact of the oral microbiota on bone activity has predominantly centered on pathogenic species and has often oversimplified disease models by overemphasizing LPS-induced osteoclastogenesis. Consequently, further studies are required to establish more physiologically relevant models and to elucidate additional regulatory mechanisms. Additionally, BEVs from periodontitis have not been routinely isolated in clinical settings. Nevertheless, *P. gingivalis* has been successfully isolated from distant tissues such as the placenta and coronary atherosclerotic plaques [150]. Given that the virulence factors of BEVs are similar to those of their parental bacteria, this discussion will focus on their virulence factors and their direct interactions with osteoblasts, osteoclasts, and immune cells.

#### 4.2.1. Virulence Factors of Oral BEVs

Oral bacterial OMVs are often enriched in virulence-associated components, and once internalized by epithelial or immune cells, these vesicles can release their cargo, including LPS and other toxic molecules, thereby triggering pro-inflammatory responses.

The fimbrial proteins that make up the fimbriae are components of BEVs, they are crucial for adhesion and possess pro-inflammatory capabilities [151]. These proteins rely on the standard TLR2 pro-inflammatory pathway involving the signal linkers TIRAP and MyD88, as well as a TIRAP/MyD88 independent pro-adhesion pathway mediated by TLR2 [42,152]. Fimbrial proteins are potentially significant in inducing bone destruction and are essential for allowing the parent bacteria to effectively infiltrate monocyte-derived DCs. However, it remains unclear whether the fimbrial proteins in BEVs assist in cell recognition by bacteria. Fimbrial proteins can prompt fibroblasts, epithelial cells, and monocyte/macrophages to produce inflammatory cytokines such as IL-1α, IL-1β, IL-6, and TNF-α. IL-1 and TNF-α are crucial local regulators in bone remodeling and play significant roles as immune mediators in periodontal inflammation and alveolar bone destruction [153].

Gingipain is a family of cysteine proteases, also known as gingipains, including arginine-specific gingipains (Rgp, including RgpA and RgpB) and lysine-specific gingipains (Kpg). In different strains of *P. gingivalis*, mature gingipains can be transported and docked on the bacterial OM or released into the extracellular environment, and have been confirmed to be enriched in *P. gingivalis* EVs [154]. Gingipains are responsible for 85% of the extracellular protein hydrolysis activity of *P. gingivalis*, by participating in the activation of host matrix metalloproteinases, stimulating oral epithelial cells to produce IL-6 and gingival fibroblasts to produce IL-8, and promoting the recruitment of polymorphonuclear neutrophils (PMN) through complement system activation, and also by cutting several TCRs and protein hydrolysis inactivation factors such as IFN-γ, IL-4, IL-5, and IL-12 to hinder host immunity, causing dysregulated immune response and inflammation [42]. Gingipains can not only regulate immune abnormalities that can cause bone loss [155], but can also directly act on osteoblasts and osteoclasts. Gingipains mediate osteoblast apoptosis in vitro experiments, possibly through two pathways: one is to inhibit the expression of integrin β1 in HCOs cell and MC3T3-E1 cell, accompanied by a decrease in active RhoA, mediating F-actin rupture and cell apoptosis [156]; another is that gingipains induce BH3 interacting domain death agonist and P53 expression upregulation by inhibiting the extracellular regulated protein kinases (ERK) and protein kinase B (PKB) pathway of MC3T3-E1 cell, causing apoptosis [157]. Although, gingipains cannot directly activate osteoclasts [158], but can upregulate osteoclast-related genes and proteins in a time-dependent manner, including cathepsin K (Ctsk), matrix metalloproteinase 9 (Mmp 9), nuclear factor of activated T-cells 1 (Nfatc 1) and tartrate-resistant acid phosphatase 5 (Acp 5), by promoting the expression of integrin αvβ, enhancing the bone resorption function of osteoclasts induced by RANKL [159], and at the same time can hydrolyze the bone protective substance secreted by osteoblasts in the extracellular [160,161], change the RANKL/OPG ratio, enhance the differentiation of osteoclasts under the induction conditions of IL-1β and TNF-α.

*T. denticola* and *T. forsythia* are important members of the red complex of periodontal disease, but unfortunately, their pathogenic mechanisms causing bone loss in periodontal disease are still poorly understood [162,163]. It is known that *T. denticola* harms include the destruction of the host cell extracellular matrix, tissue infiltration, and destruction of the host cell membrane [164,165], accompanied by the dysregulation of host immune regulatory factors [166]. *T. forsythia* produces inflammatory signals through TLR2, leading to alveolar bone loss related to Th2 cells, among which, BspA protein not only plays a role in adhesion, but also triggers the release of pro-inflammatory cytokines from monocytes and chemokines from osteoblasts, leading to inflammation and bone resorption [167]. However, the pathogenicity of *T. forsythia* depends on the role of other bacteria, and bone loss cannot be observed in GF mice. Interestingly, although the BEVs of both induce Th cells polarization in bone-marrow-derived dendritic cells (BMDCs), the mechanism is complex, *T. denticola* EVs mediate Th17 polarization, while *T. forsythia* EVs mediate Th1 polarization, which is inconsistent with the result of *T. forsythia* GroEL cooperating with IL-17 to induce bone loss, which may be related to different BEVs enrichment components [168]. Proteases (such as dentin) on *T. denticola* EVs widely hydrolyze cells to produce IL-1β, IL-6, IL-8, and TNF-α. The S-layer glycan structure on *T. forsythia* EVs protects parent bacteria from being recognized by BMDCs [169]. Therefore, the mechanism of immune dysregulation caused by these BEVs may be more important in bone loss.

*F. nucleatum* is also a rich periodontal bacterium, present in the oral cavity of both periodontally healthy and diseased patients [170]. *F. nucleatum* EVs contain a variety of antigenic virulence factors, including FadA, MORN 2, and YadA-like domains [171]. The virulence factor FadA allows *F. nucleatum* to survive under stress conditions, form biofilms, accumulate in diseased periodontal sites, bind to host cells, and induce periodontal bone loss. However, amyloid-like FadA does not seem to independently cause the manifestation of OP/bone loss, but enhances the virulence potential of *F. nucleatum*, because *F. nucleatum* lacking FadA did not detect significant periodontal bone loss [170]. Although, FadA has the function of regulating cell Wnt/β-catenin [172], but no abnormal function of bone lineage cells has been found yet. But some researchers have found that FadA plays an important role in the pathogenesis of RA, which will be discussed later.

#### 4.2.2. Possible Role of Oral BEVs and Osteoporosis

While research has well-established the direct relationship between bacteria and alveolar bone resorption in the oral pathological microenvironment, the association of BEVs with systemic bone loss requires more direct evidence. Six groups of bacteria species are highly associated with biofilm formation and maturation, contributing to periodontal bone loss. Among these, the ‘Red complex,’ which forms at a later stage, is associated with the progression of periodontitis. This complex comprises *P. gingivalis*, *T. denticola* [165,173,174], and *T. forsythia* [175,176], and could release EVs into oral environment. Under the dual stimulation of dysregulated microbiota and excessive immune response, the homeostasis of alveolar bone tissue is disrupted, leading to bone resorption and progression of periodontitis. Due to their nanoparticle size, adhesiveness, and proteolytic properties, BEVs can migrate through host tissues, disrupt tight epithelial connections, and deliver bacterial virulence factors to immune cells in the underlying tissues.

BEVs derived from *Filifactor alocis* (*F. alocis*) inhibit the differentiation and mineralization of BMSCs osteoblasts in a dose-dependent manner, leading to downregulation of the alkaline phosphatase, osteocalcin, and type I collagen [23]. BEVs modulate the MAPK NF-κB signaling pathway via TLR2, which leads to reduced expression of osteogenic marker genes during the osteogenic differentiation process of BMSCs, thereby inhibiting osteogenic differentiation [177,178,179].

**Figure 3:**
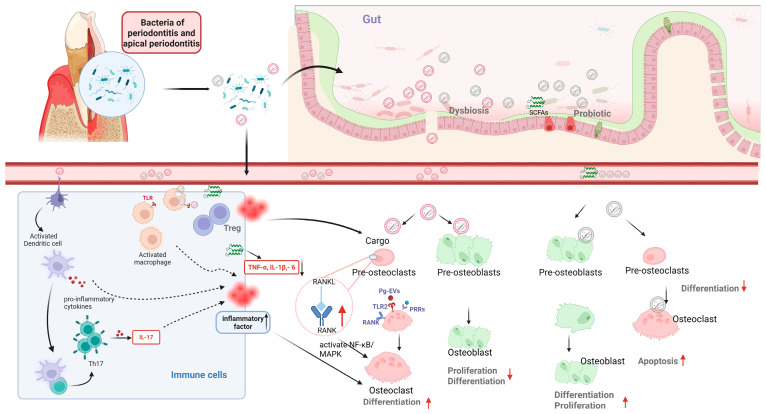
Schematic representation of how oral BEVs from periodontitis and apical periodontitis translocate to the gut, contribute to dysbiosis, and subsequently reach the circulation to modulate bone homeostasis. BEVs activate dendritic cells and macrophages through PRRs such as TLRs and NOD1, inducing Th17 responses and IL-17/RANKL signaling that drive osteoclastogenesis. In parallel, BEVs can directly interact with pre-osteoclasts via TLR2/RANK pathways to enhance NF-κB/MAPK/NFATc1 activation, while impairing osteoblast proliferation and differentiation. Conversely, commensal-derived metabolites such as SCFAs promote Treg expansion and exert protective effects against excessive inflammation and bone resorption. Pathways depicted in this figure were adapted and summarized from previously published studies [141,147,185,186,187,188,189]. Created in Biorender. (2025).

In addition to inhibiting osteogenic processes, BEVs also facilitate osteoclastic bone resorption by promoting osteoclastic activation and differentiation, inducing macrophage polarization, and facilitating the release of inflammatory mediators, as depicted in Figure 3. (1) *F. alocis* EVs predominantly activate the TLR2 pathway, leading to the phosphorylation of NF-κB, ERK, JNK, and p38, which in turn stimulates the expression of osteoclastogenic transcription factors NFATc1 and c-Fos. While other TLRs like TLR4, TLR9, or the intracellular receptor NOD has also been reported [23,180], TLR2 appears to be the primary immune receptor for BEVs on the cell surface. The effectiveness of BEVs on osteoclast differentiation is notably reduced in TLR2-deficient osteoclast precursors [25], which is associated with abundance of LPS and lipoproteins in BEVs. (2) *F. nucleatum* EVs effectively recruit macrophages to the alveolar bone, promoting their differentiation into a pro-inflammatory M1 phenotype in a time- and dose-dependent manner. This increases the release of IL-1β, IL-6, and TNF-α, driving bone resorption during the early stages of disease [181,182]. The polarizing effect of *F. nucleatum* EVs on macrophages remains a subject of debate, with some studies suggesting a potential role in M2 polarization, indicating that further verification is needed [183]. (3) BEVs stimulate macrophages, DCs, epithelial cells, endothelial cells, and others to secrete pro-inflammatory factors such as TNF-α, IL-1, and IL-6. Note that the immune responses were dependent on EVs concentration. At low concentrations, these EVs provoke the secretion of TNFα, IL-1β, and IL-8 in unprimed cells, recruit inflammatory cells in vivo, and trigger macrophages to produce a heightened immune response upon a second exposure. Conversely, high concentrations of *P. gingivalis* EVs induce less inflammation initially due to LPS tolerance and inflammatory unresponsiveness, but lead to strong inflammasome activation upon subsequent exposure. This flexible immune stimulation might facilitate manipulation and disruption of host immune responses, potentially accelerating disease progression alongside pathogens such as *T. denticola* and *T. escherichia* [154,184].

### 4.3. Oral/Gut BEVs and Arthritis

RA is an autoimmune disease affecting nearly 1% of the world’s population. It is characterized by high levels of serum inflammatory cytokines, including IL-6 and TNF-α, triggering inflammatory processes in the joints, leading to progressive joint destruction and physical disability. However, the potential mechanisms of the origin of inflammation have not yet been determined. Notably, multi-omics analysis of fecal, serum, and synovial fluid samples from RA patients indicates that the microbiota, through metabolic secretion and microbial invasion, is closely related to the severity of RA.

Hong et al. observed in collagen induced arthritis (CIA) mice that BEVs containing *F. nucleatum* virulence determinant FadA translocated to the joints, triggering a local inflammatory response. Specifically, FadA acts on synovial macrophages, leading to the activation of Rab 5a GT3 and YB-1 (regulator of inflammatory mediators) involved in vesicle transport and inflammatory pathways, thereby triggering and exacerbating joint inflammation. Most importantly, the downstream signal FadA-Rab 5a-YB-1 of *F. nucleatum* EVs was confirmed in RA patients, indicating that *F. nucleatum* induces the occurrence of RA by secreting EVs containing FadA [190].

Citrullinated proteins play an important role in the pathogenesis of RA. In the early course of RA, the appearance of anti-citrulline antibodies (ACPA) and anti-citrullinated fibrinogen antibodies (ACF) suggests that joint inflammation will be more severe in antibody-negative patients, and the loss of tolerance to citrullinated proteins in RA patients exacerbates the development of the disease. The peptidylarginine deiminase (PPAD) of *P. gingivalis* is an enzyme that can citrullinate human proteins and may cause RA patients to lose tolerance to citrullinated proteins [191,192,193]. Other researchers believe that truncation of the active PPAD N-terminus may prevent citrullination, and citrullination of PPAD as a link between periodontal disease and RA is not rigorous [194]. In short, PPAD is a risk factor worth exploring. Recent research further confirms that PPAD is retained in the OM of *P. gingivalis* cells and is secreted into the extracellular environment, where PPAD is detected in a soluble form and associated with BEVs [195]. Gabarrini et al. confirmed the importance of A-LPS modification in the association of PPAD with OM and BEVs, and BEVs protects A-LPS-modified PPAD from protease degradation [196]. Although the relationship between PPAD and BEVs and the mechanism of citrullination of peptidylarginine suggest the possibility of PPAD pathogenesis in RA. At present, there are few articles that explore the structure of PPAD in BEVs and the possible pathogenic mechanism in RA from the perspective of BEVs more cautiously.

In addition, neutrophils are trapped in a vicious cycle in RA, which may be related to the regulation of neutrophils by pathogenic BEVs. The synergistic effect of macrophages and neutrophils on RA has received much attention in recent years [197]. RA is a risk factor for Staphylococcus aureus bacteremia (SAB). *P. gingivalis* EVs help *S. aureus* internalized into neutrophils, and translocated to the bloodstream through neutrophil translocation [198], while simultaneously activating more neutrophils [199] and causing further aggravation. We have summarized the recent dosage and frequency of medications used for bone-related disorders in BEV manufacturing systems in Table 1 for reference.

In summary, the relationship between oral–gut BEVs and bone diseases is vague and complex, which is related to the variable microbial environment in the body and the unstable composition of BEVs components. Multiple sources of BEVs coexist, and there may be both competition and mutual benefit in regulating the function of host cells. Finding the key regulatory factors of BEVs in the microbial community in bone diseases may be the focus of future research.

## 5. New Possibilities for Oral–Gut BEVs in Regulating Bone Homeostasis

The connection between oral–gut bacteria and local to systemic bone immunity has been extensively studied [29,53,207], and changes in the microbiota, immune cells, and immune factors as new characteristics of bone homeostasis may affect the treatment strategies and prognosis of bone diseases. Given that BEVs contain bacterial structural components and can transport bacterial products, they represent a critical conduit through which oral–gut microbiota may influence bone homeostasis via distal bone immunity. The role of inflammation and anti-inflammatory responses in bone immunity has gradually attracted attention to the important role of oral–gut BEVs in bone immunity [117,208,209]. Further research on the mechanism of action of BEVs will help understand the regulatory mechanism of the microbiota in bone homeostasis. Beyond their recognized impact on bone immunity, BEVs are also central to emerging research on the endothelial–bone and neural–bone axes. The vascular system and nervous system in the skeletal system play an indispensable role in the physiological and pathophysiological processes of the skeletal system [210,211]. Empirical studies, both in vivo and in vitro, have documented the effects of BEVs on endothelial cells and neurons, suggesting novel regulatory pathways: the BEVs–endothelial–bone axis and the BEVs–neural–bone axis. Therefore, we propose two new pathways for oral enterobacterial BEVs to regulate bone homeostasis: the BEVs–endothelial–bone axis and the BEVs–neural–bone axis.

### 5.1. Oral–Gut BEVs and the Endothelial–Bone Axis

Blood vessels, consisting of a well-organized system of endothelial tubules, are critical for bone tissue oxygenation, metabolism, and immune surveillance. The critical role of angiogenesis and different capillary subtypes in bone development, homeostasis, and repair has been extensively reviewed [210]. These vascular cells are not just building blocks of the transport network, but actively control key processes through communication with various other cell types, including interactions among chondrocytes, perivascular osteoblast lineage cells, macrophages, and osteoclasts.

BEVs derived from intestinal *E. coli* have been shown to upregulate the expression of endothelial cell adhesion molecules ICAM-1 and VCAM-1 [212], thereby initiating inflammation. Furthermore, BEVs from *P. gingivalis* have been found to induce dysfunction in human retinal microvascular endothelial cells, leading to increased ROS production, enhanced endothelial permeability, and apoptosis [213,214]. Jia et al. reported that *P. gingivalis* EVs trigger the activation of Rho-associated coiled-coil forming protein kinase (ROCK) in a time- and dose-dependent manner, causing various biological effects such as cytoskeletal changes that disrupt cell secretory properties, contribute to endothelial dysfunction, and promote vascular smooth muscle cell proliferation. This, in turn, inhibits the expression levels of endothelial nitric oxide synthase (eNOS) in aortic endothelial cells [215]. Additionally, Bartruff et al. found that *P. gingivalis* EVs dose-dependently inhibit the proliferation of HUVECs and the formation of capillary tubules [216]. Given that the progression of RA is characterized by endothelial dysfunction [217], further investigation into the impact of BEVs on endothelial cells could provide valuable insights into their role in RA and potentially other vascular-related bone diseases. Recent research has linked bone marrow inflammation to the growth of type H blood vessels and associated pathological osteogenesis [218]. Since a decrease in the level of the cell surface intercellular adhesion molecule CD31 in HMECs was observed under the influence of *P. gingivalis* gingipain-related EVs [74], suggesting possible role of BEVs on endothelial cells on bone metabolism might operate through vascular functions.

### 5.2. Oral–Gut BEVs and Neuro–Bone Axis

Over the past two decades, the role of intraosseous nerves in bone has emerged as a significant research topic. The interaction between nerves and bones extends beyond physiological processes to encompass various bone diseases, including OP and osteoarthritis. Recent findings have drawn attention to the pathological effects of nervous system changes on bone conditions. Neurodegenerative diseases, often resulting from prolonged neural inflammation, lead to neuronal degeneration and death, as seen in AD, Parkinson’s disease (PD), and Huntington’s disease. Epidemiological studies have indicated that neurodegenerative changes, particularly those characteristic of AD, often coincide with OP in the elderly population [219]. Research by Sameet et al. suggested that individuals with AD exhibit compromised bone health compared to their age-matched peers [220,221]. Loskutova et al. reported that early AD patients observed a decrease in the volume of the hypothalamus and multiple brain regions, including cognitive brain structures, such as the hippocampus and cerebral cortex, these areas are related to the regulation of bone throughout the body [222,223]. Further, studies in hTau mouse models by Dengler-Crish et al. showed that a decrease in serotonergic neurons early in AD correlates with reduced bone density [224]. Similarly, Parkinson’s model mice have demonstrated bone loss associated with the degeneration of dopaminergic neurons [225,226,227]. Additionally, patients with head injuries or spinal cord injuries exhibit abnormal callus formation and significantly lower bone density below the lesion site compared to controls, underscoring the potential role of impaired neural function in abnormal bone metabolism [228].

Clinical [20,229,230] and basic research [50,231] evidence both support the close relationship between gut bacteria and nerve lesions. Elefteriou et al. employed pseudorabies virus (PRV) to trace neuronal circuits linking the central nervous system with the skeleton, revealing active connections across brainstem, pons, midbrain, hypothalamus, and sacral spinal cord segments to the spinal cord, brain, and femoral nerve fibers. This supports the notion that nerve damage may contribute to bone loss in broader systemic diseases [232]. This confirms the possibility of nerve damage leading to bone loss in macroscopic diseases. Central nervous system damage is often accompanied by various types of neuroaxis dysfunction [233]. Abnormal regulation of the hypothalamic–pituitary–adrenal axis leads to disorders of hormone homeostasis such as NPY, neuropeptide U, follicle-stimulating hormone, glucocorticoids, etc., affecting bone metabolism [234]; autonomic nervous system dysfunction leads to related neurotransmitter dysfunction, such as parasympathetic nucleus injury leading to a decrease in parasympathetic tension, and a decrease in acetylcholine (Ach) secretion in the bone marrow cavity leads to an increase in the number of osteoclasts, leading to bone loss [232,235]; disorders of the enteric nervous system lead to the loss of gut neurons, immune disorders and increased gut barrier permeability, leading to a vicious cycle of gut flora disorder, which is often not conducive to bone health [233].

Research indicate that *P. gingivalis* EVs can cross the BBB [94,226] and translocates to the brain [120,226,236,237], initially triggering microglial immune responses and escalating brain inflammation dose-dependently. High levels of inflammatory factors can lead to amyloid plaques, Tau hyperphosphorylation, and myelin damage [236,238,239,240]. BEVs from alkaline-producing bacteria in the gut cause severe enteritis, infiltrate hippocampal neurons through the bloodstream and vagus nerve [241], and induce memory loss and cognitive impairment in model mice [94], highlighting the adverse impact of BEVs on neuronal function.

The peripheral nervous system plays a vital role in regulating bone homeostasis through various neurotrophic factors [242]. The activation of nociceptors, which involves neurons from the nodose ganglion, trigeminal neurons, and dorsal root ganglion, is notably linked to bone loss. When these nociceptor neurons are exposed to excessive peripheral nociceptive stimuli, they become sensitized. LPS on BEVs may activate sensory neurons, causing an influx of calcium ions directly through the TLR4 receptor, or alternatively, mediates pain through the non-TLR4-dependent nociceptive ion channel TRPV1. Moreover, these nociceptor neurons express TLRs and respond to PAMPs containing BEVs. This interaction induces the upregulation of inflammatory mediators, including prostaglandin E2 (PGE2) and IL-1, which amplify the inflammatory response, enhance TRPV1^+^ afferent signal transmission, and ultimately promote bone loss [243,244].

In summary, oral–gut BEVs are closely related to many neurological diseases, including autism, anxiety, obesity, schizophrenia, PD, and AD. The modulation of the nervous system by BEVs represents a potential mechanism influencing bone homeostasis. Recently, intraperitoneal administration of *P. gingivalis* EVs in mice was found to induce osteoporosis accompanied by cognitive dysfunction. Accumulation of EVs was observed in both brain tissue and long bones, while in vitro experiments demonstrated downregulation of acetylcholine secretion in neuronal cells, which may contribute to bone loss. Nevertheless, further clinical studies are required to validate these findings and identify potential clinical correlates [27]. Investigating the regulatory role of BEVs within the neuro-bone axis could not only assist in preventing bone-related complications in patients with neurological diseases, but also offer innovative approaches for bone neurotherapy.

## 6. Conclusions

Oral- and gut-microbiota-derived BEVs have emerged as critical mediators of host–microbe interactions that extend beyond local niches to influence systemic bone homeostasis. Current evidence highlights that pathogenic oral microbiota derived BEVs contribute to periodontal disease and may play roles in systemic bone disorders; however, rigorously controlled animal models and clinical studies are urgently needed to substantiate these links. The heterogeneity of BEV cargo, shaped by environmental factors such as pH and vesicle size, together with size- and surface-dependent uptake mechanisms, introduces variability but also reveals novel opportunities. Understanding these mechanisms will be essential to advance both mechanistic insight and therapeutic applications.

## 7. Limitation and Future Prospects

Despite the growing interest in BEVs, several limitations remain. Current research often focuses on BEVs from single bacterial species, while the pathogenesis of diseases such as periodontitis is driven by polymicrobial communities. In this context, pathogens actively disrupt host immune responses, creating a cycle of tissue destruction and immune dysregulation that sustains local inflammation and contributes to systemic complications. By contrast, experimental models using injected BEVs may idealize their effects, exaggerating the role of a single vesicle population while overlooking the complexity of host–microbe interactions at the systemic level. To move forward, emerging technologies such as spatial transcriptomics and single-cell multi-omics will be essential for precisely mapping BEV–host interactions within defined tissue microenvironments.

On the translational front, BEVs offer both opportunities and challenges.

Opportunities: BEVs possess the unique ability to cross biological barriers and deliver therapeutic cargo to distal tissues, thereby improving drug delivery efficiency for bone and neurodegenerative diseases. Engineering approaches integrating osteogenic factors such as BMP-2 and CXCR4 have been used to develop active targeting strategies with synergistic effects [203]. Similarly, recombinant BEVs displaying the pre-osteoclast fusion protein DC-STAMP and loaded with FRAT have demonstrated potent bone-targeting and therapeutic efficacy [204]. BEVs can also be incorporated into biomaterials to achieve sustained osteogenesis and angiogenesis in osteoporotic fracture models [245]. Beyond acting as delivery systems, BEVs themselves may serve as therapeutic agents; for example, commensal BEVs have been shown to inhibit pathogenic bacteria, modulate immunity, and prevent disease progression [97]. Importantly, BEVs are generally safer than live bacteria, reducing risks of uncontrolled colonization and infection.

Challenges: Several critical hurdles remain before BEVs can be translated into clinical practice. First, the intrinsic heterogeneity of BEV cargo, shaped by environmental cues such as pH and bacterial growth phase, complicates reproducibility. Addressing this requires advanced isolation and sorting technologies with improved yield, purity, and the ability to separate contaminants. Second, engineering BEVs for safety and efficacy is a priority, particularly the need to reduce or eliminate immunogenic components such as OmpA or excessive LPS, while preserving desirable adjuvant properties (e.g., low-level LPS for vaccine use). Third, the mechanisms of cellular uptake remain incompletely understood. For instance, strategies that bias uptake toward osteoblasts or BMSCs—while minimizing phagocytic clearance—could improve the therapeutic efficiency of BEV-based miRNA delivery. Similarly, the molecular basis of BEV transport across the blood–brain barrier remains poorly defined. Finally, challenges of stability, scalability, and industrial production must be addressed through advances in bioprocessing and formulation.

The convergence of nanotechnology, bioengineering, and systems biology may enable the rational design of BEVs with tailored surface modifications, optimized cargo, and enhanced stability. Such advances will not only accelerate therapeutic applications but also position BEVs as next-generation tools for diagnosis, prevention, and treatment of bone-related diseases.

## Figures and Tables

**Table 1:** Summary of preclinical animal studies investigating the roles of BEVs in bone- and inflammation-related diseases, as well as probiotic- or engineered-BEV-based interventions in disease models. The table outlines the bacterial source, dosage/concentration, route and frequency of administration, disease models, and proposed mechanisms of action.

BEV Source	Dosage/Concentration	Route of Administration	Dosing Frequency and Duration	Disease Model	Mechanism	Animal Model	Ref
*Porphyromonas gingivalis*	50 µg	i.p., oral gavage	Every 2 days for 14 days	Osteoporosis/cognitive dysfunction	Inhibition of Ach secretion of neuron cells	C57BL	[188]
*Aggregatibacter actinomycetem-comitans*	2.25 × 10^9^ particles	Gingiva injection	Once	Periodontitis/trigeminal ganglion neuroinflammation	Cross BBB and increase pro-inflammatory cytokine expression	C57BL/*MyD88*^−^ mice	[200]
*Fusobacterium nucleatum*	2.36 × 10^9^ particles	Gingiva injection	3 times per week for 4 weeks	Periodontitis	Promote local inflammation	SD	[201]
*Filifactor alocis*	6.0 × 10^11^ particles	i.p.	Days 0 and 3, for 7 days	Osteoporosis	Increase osteoclast-activating cytokines expression through TLR2 signaling	C57BL/6	[23]
*B. acidifaciens*	50 μg	Oral gavage	Once every day for 10 days	Colitis	Reduced the expression of pro-inflammatory genes, and enhance the epithelial barrier, stabilizing the distribution of intestinal mucin proteins.	C57BL/6	[202]
Engineered BEVs-BMP-2-CXCR4	0.5 µg/µL	Tail vein injection	Once a week for 8 weeks	Osteoporosis treatment	Target BMSCs in bone marrow, thereby promoting osteogenic differentiation and inhibiting their adipogenic differentiation via BMP/SMAD signaling pathway	C57BL/6	[203]
EcN-pClyA-DC-STAMP	5 mg/kg	Tail vein injection	Once a week for 7 weeks	Osteoporosis treatment	FRAT bind the phosphorylated GSK3β and regulate the Akt/GSK-3β/NFATc1 signaling pathway for inhibiting the formation and bone resorption of osteoclasts	C57BL/6	[204]
*Lactobacillus salivarius*	50 µg/mL	Oral gavage	Once every other day for 8 weeks	Osteoporosis treatment	EVs of *L. salivarius* could be transported to the bones and prevent ORX-induced osteoporosis	Sprague Dawley	[205]
*Escherichia coli* Nissle 1917	5 × 10^9^ CFU	Oral gavage	Every two days for 28 days	Hyperuricemia treatment	Enzyme-loaded OMVs also effectively detoxify human serum samples	Kunming rat	[206]

i.p.: intraperitoneal injection; oral gavage.

## Data Availability

No new data were created or analyzed in this study. Data sharing is not applicable to this article.
